# Robust Fall Army Worm detection in maize using multimodal RGB and thermal image fusion

**DOI:** 10.1038/s41598-025-29784-8

**Published:** 2025-12-25

**Authors:** Prakash Sandhya, B Venkataramana

**Affiliations:** Department of Mathematics, School of Advanced Sciences, Vellore Institute of Technology, Vellore, Tamil Nadu India

**Keywords:** Image Fusion, FAW, Infrared / Thermal image, Visible / RGB image, ViT, CNN, Plant sciences, Computational biology and bioinformatics, Data integration, Image processing, Machine learning

## Abstract

Effective pest and disease detection plays a crucial role in minimizing crop losses and improving decision-making in precision agriculture. Among the most destructive pests affecting maize crops globally is the Fall Army Worm (FAW), known for its rapid spread and high impact on yield. Existing detection practices often rely on manual scouting, which can be inefficient, labour intensive and prone to human error. This study proposes a novel deep learning based framework for the automatic classification of FAW infested and healthy maize crops by integrating RGB and thermal image modalities. The core objective is to enhance detection accuracy through multimodal image fusion. A hybrid DNN-ViT model is introduced, combining two complimentary pipelines: (i) feature-level fusion, where CNN extracted features from RGB and thermal images are fused and classified using a Deep Neural Network (DNN) and (ii) image-level fusion, where a 6 channel RGB-thermal image is directly processed using a modified Vision Transformer (ViT). Experimental results demonstrate that the fused model achieved superior performance with an accuracy of 0.98, precision, recall and F1-score of 0.98 and AUC-ROC of 0.98 on the test set, outperforming models trained on RGB-only, thermal-only and unfused data. The ablation study confirms the effectiveness of multimodal fusion, with the no-fusion model showing significantly lower performance (accuracy-0.60 and AUC-ROC-0.67). This work highlights the benefits of integrating complementary data sources for robust crop health monitoring. Future research will explore enhanced fusion strategies, environmental robustness and field level deployment to validate the model’s practical applicability.

**Supplementary Information:**

The online version contains supplementary material available at 10.1038/s41598-025-29784-8.

## Introduction

Pests continue to be a major challenge to global agriculture, causing an estimated 40% loss in crop yield annually, equivalent to approximately 70 billion USD in economic damage^1^. Effective pest management, particularly in staple crops like maize, relies heavily on early, accurate identification and classification of infestations. Among these pests, FAW emerged as a devastating invasive species that severely threatens maize production worldwide. Timely detection is crucial to enable targeted interventions, minimize crop loss, reduce pesticide overuse and enhance overall yield and sustainability. Conventional pest detection techniques, which are primarily based on manual scouting and visual inspection, are labour intensive, prone to human error, and often delayed. Moreover, traditional image-based detection methods that rely solely on RGB imagery are limited by environmental factors such as lighting conditions and occlusions^2^. As a result, researchers have increasingly turned to multimodal sensing approaches, such as combining RGB and thermal imaging, to leverage complementary strengths.

Image fusion techniques aim to integrate information from multiple sensors to produce a more informative composite image that improves both human perception and machine analysis^3–5^. Fusion of visible and infrared images, in particular, offers synergistic advantages: visible images provide fine texture and structural detail under adequate lighting, while infrared (thermal) images capture thermal radiation and remain effective under low visibility and adverse weather conditions^6–11^. This complementary information can be leveraged through fusion techniques at the pixel, feature, or decision level^12–25^.

Recent advances in deep learning have transformed image fusion, enabling more robust and adaptive models. Convolutional Neural Networks (CNNs) have been widely used for segmentation and feature extraction tasks, particularly when input images are registered^18,26–32^. However, CNNs primarily capture local features, often at the cost of losing global spatial relationships^33^. Vision Transformers (ViTs), with their self-attention mechanisms, are capable of modeling long-range dependencies and have shown promise in various computer vision tasks^34,35^. Yet, most transformer-based methods are designed for single-modality data and are rarely applied in the context of infrared-visible image fusion for pest (FAW) detection.

This study introduces a novel approach that integrates feature level fusion of RGB and thermal images with a hybrid deep learning architecture combining a Deep Neural Network (DNN) and ViT. Unlike conventional fusion pipelines that rely on pixel level fusion or single modality processing, our method performs registration-free feature-level fusion, allowing the model to directly handle potentially misaligned sensor inputs. This is particularly useful for in-field agricultural imaging, where perfect alignment between sensors is difficult to achieve. The fused features are then classified using ViT, leveraging its global attention capabilities to improve classification performance.

Our key hypothesis is that the complementary nature of RGB and thermal modalities provides a more comprehensive representation of crop health, leading to improved detection of FAW infestation. This work not only explores an underutilized direction in pest detection-multimodal fusion using transformer based architecture, but also contributes a scalable solution for practical deployment in precision agriculture. The rest of the paper is organized as follows: Section “Related Literature” reviews related literature on image fusion and pest detection. Section “Methodology” describes the proposed methodology, including the dataset acquisition, feature level fusion strategy and model architecture. Section “Discussion” provides a detailed discussion of the findings, including an ablation study and visualization analysis. Finally, Section “Conclusion” concludes the paper and outlines the directions for future research.

## Related literature

Combining thermal and RGB images has become a key focus in computer vision research, with applications in diverse fields like surveillance, autonomous driving, medical imaging, and search and rescue. Thermal images capture infrared radiation, providing crucial information in low-light or obscured conditions, while RGB images offer detailed visual information within the visible spectrum. Fusing these modalities significantly improves image quality and enhances the robustness of vision systems, especially in complex and dynamic environments.

Early thermal and RGB image fusion relied on classical image processing methods like wavelet transforms, multi-resolution analysis, and pixel-level fusion^3–5^. While effective in some cases, these methods often struggle to capture complex patterns and spatial relationships within the data. Additionally, they have difficulty preserving fine features and managing input misalignments or noise. The advent of deep learning (DL) has revolutionized image fusion by enabling the learning of intricate data representations. Convolutional Neural Networks (CNNs), through automatic hierarchical feature extraction, became foundational in fusing RGB and thermal imagery. CNN-based models can extract both spatial and semantic information efficiently, supporting multiple input sources.

Recent research reviewed in^36^ provides both qualitative and quantitative evaluations of current deep learning-based fusion algorithms. A novel deep neural network architecture, FNSTC, which integrates ConvNeXt and Swin Transformer backbones, was shown to yield remarkable performance by fusing features early, combining the strengths of both convolutional and transformer-based approaches^1^. An MRF-based image fusion method^13^ balances local saliency and global smoothness, solving a Poisson equation to reconstruct the fused image. Another MRF based approach uses a maximum a posteriori (MAP) framework for effective structural preservation^4^.

In agriculture, wavelet image fusion was employed for early pest detection^37^, while Pest-YOLO combined YOLOv4 with attention-enhanced CNNs to detect small pests effectively^2^. These studies demonstrated the potential of image fusion for field-level decision-making support. Recent studies^38^ proposed a modified ResNeXt model for the classification of fungal crop diseases using RGB imagery from heterogeneous regions. The model effectively handled data from apples, guavas and custard apples achieving high accuracy in disease detection. Further enhancements were introduced in^39^, where an improved version of ResNeXt incorporated preprocessing and segmentation to refine region-of-interest extraction in apple disease classification. Additionally^40^, presented a segmentation-based approach, SegLearner, that quantified disease severity using pixel-level analysis. This model bridges classification and spatial interpretation, offering critical insights into the extent of infection—an essential requirement for precision agriculture. CNN based models continue to dominate fruit disease classification, with recent studies demonstrating high accuracy (up to 99.2%) for potato leaf diseases such as early and late blight. These models often leverage pretrained backbones like VGG19 for feature extraction and fine-tuning showing their effectiveness even in relatively constrained datasets^41^.

Sparse representation-based fusion methods also advanced, including a local contrast preprocessing strategy to preserve important image details^42^ and a K-SVD based supervised classification fusion framework for improved sparse coding^5^. Spectral Edge fusion, a technique originally developed for RGB-NIR integration, was adapted for smartphone-based RGB-thermal fusion, highlighting its flexibility^43^. A gradient-domain fusion method based on MRFs was introduced to optimize both local and global image structures^13^. Hybrid CNN-Transformer models have now gained attention due to their combined ability to preserve local detail (CNNs) and capture global context (Transformers), illustrated using windowed gait energy images to optimize feature learning^28^.

For robust object representation, a target-enhanced MST method based on Laplacian pyramids weighted by infrared information allowed user-defined target emphasis, enhancing fusion quality^15^. Similarly, a rolling guidance filter-based method enhanced thermal information and visible detail but faced challenges in manual parameter tuning and computational load^6^. A multi-scale parallel cross-fusion network (MPCFusion) efficiently extracted deep and modality-specific features using cross attention mechanisms, effectively, improving information exchange between modalities^44^.

Other studies addressed misalignment and domain differences. A pixel-level fusion strategy was proposed for vision-based relative navigation, addressing the illumination sensitivity of RGB images by integrating thermal infrared features^12^. A position-unaware style fusion loss function was trained with an unsupervised network, aligning feature distributions of thermal and visible domains^11^. CUFD, another novel approach, used dual encoder-decoder architectures for decomposing and fusing common and unique modality components^16^. In the NSCT domain, fusion schemes inspired by the human visual system helped guide optimal low- and high-frequency sub-band selection^7^. In the medical domain, PDRF-Net a progressive dense residual fusion network, demonstrated high performance for COVID-19 lesion identification in CT scans^29^. It incorporated dense skip connections and aggregated residuals for improved segmentation. The NSCT-VGG-Net method further fused thermal and visible images by combining NSCT with deep features from VGG19^17^.

For surveillance, MDA (Multiscale Dual Attention) used attention blocks and novel loss terms to preserve both thermal and textural data^9^. FusionGAN adopted an adversarial framework to generate high-quality fused images, working even with different image resolutions^25^. GANMcC addressed fusion as a multi-distribution estimation problem, using classification constraints to balance thermal and visual contributions^45^. SimpleFusion, based on Retinex theory, decomposed images into reflectance and illumination using lightweight CNNs and dual loss terms, achieving high performance in preserving complementary modality information^10^. CMFuse introduced intra- and inter-modal attention mechanisms via MLPs, improving local fine-grained detail retention^23^.

In remote sensing, SwinFuSR enhanced thermal image resolution with RGB guidance and overcame registration errors^46^. STSR generated high-resolution satellite imagery by addressing spectral shifts using fuzzy classification and object-based constraints^19^. For real-time applications, AGMFusion introduced adaptive guidance and content loss to improve perceptual quality while maintaining lightweight deployment^3^. The double-channel image fusion method combined self- and cross-attention with a focusing loss to enhance detail preservation across varied scenes^26^. In infrastructure monitoring, RGB-thermal fusion using ResNet enabled robust damage detection even in low-light settings^22^. A Chan-Vese-based registration model facilitated fruit defect detection through fusion^32^. In multispectral object detection, fused RGB-LWIR models from airborne sensors improved edge detection and target recognition, even in low-visibility conditions^47^.

SimpliFusion, a lightweight transformer model, integrated spatial–temporal features and modality-specific details for high-quality image fusion^21^. CTHIE (CNN-Transformer Hybrid for Image Enhancement) aligned misaligned images without explicit registration, demonstrating efficacy on real and synthetic datasets^35^. RITFusion, a reinforced interactive transformer with skip connections and morphological loss, improved fusion for challenging inputs^48^. SFPN (Semantic Fusion Pyramid Network) extracted multi-scale features for multi-focus image fusion using segmentation-aware mechanisms^18^. MST-based segmentation incorporated human vision and spatial factors into a novel pixel-weighing strategy for better segmentation^30^. GAN-based fusion using NSCT residuals^49^ and ViT-CNN hybrid models demonstrated that combining attention and frequency based methods can improve both feature completeness and visual appeal. Interactive self-supervised networks for fusion were introduced to retain key multi-modal information across hierarchical levels^50^.

To address the limitations of conventional segmentation methods under complex natural scene variability, hybrid techniques such as FCPN (Fuzzy Competitive Learning-based Counter-Propagation Networks) have shown promise. This method leverages both fuzzy logic’s uncertainty handling and neural networks’ learning capabilities, achieving high accuracy and automatic cluster estimation for image segmentation tasks^51^. Drone-based imaging combined with computer vision has emerged as a powerful tool in agriculture for crop monitoring, weed mapping, and pest detection. Recent comprehensive surveys emphasize that, despite promising applications, real-world deployment remains hindered by a lack of technical know-how and infrastructure support. Studies provide detailed taxonomies covering drone types, sensors, vegetation indices, processing software, and legal challenges, highlighting the scope and potential impact of these technologies on yield improvement and farmer empowerment^52^. Recent work on Cherimoya leaf disease classification compared five deep learning models:EfficientNet, MobileNetV2, BiT, EANet and Swin Transformer-demonstrating that EANet achieved the highest accuracy with 96.89% test accuracy and an F1score of 0.98, underscoring the importance of choosing suitable architectures tailored to plant species-specific image characteristics^53^.

Overall, deep learning architectures have greatly advanced the capabilities of thermal-RGB image fusion for diverse real-world applications. However, challenges remain in handling data misalignment, preserving fine features across modalities, and minimizing dependency on large annotated datasets. Despite promising results, hybrid architectures that intelligently combine the strengths of CNNs and Transformers, along with domain specific loss functions, present the most potential for advancing RGB-thermal image fusion in precision agriculture and beyond. In agriculture, although fusion has been used for tasks like fruit defect detection and pest segmentation, no existing studies focus specifically on FAW in maize, particularly using field-compatible thermal sensors such as the FLIR infrared camera. Moreover, many prior models either assume pixel-perfect registration or rely on computationally heavy architectures that are unsuitable for real-time deployment in field conditions. This study addresses these challenges by proposing a registration-free feature level fusion method integrating RGB and thermal modalities through a hybrid DNN-ViT architecture. The proposed approach is explicitly tailored for the early detection of FAW under practical field constraints, thus filling a critical gap in precision pest monitoring systems using deep learning-based multispectral fusion.

## Methodology

### Dataset and preprocessing

**Fig. 1:**
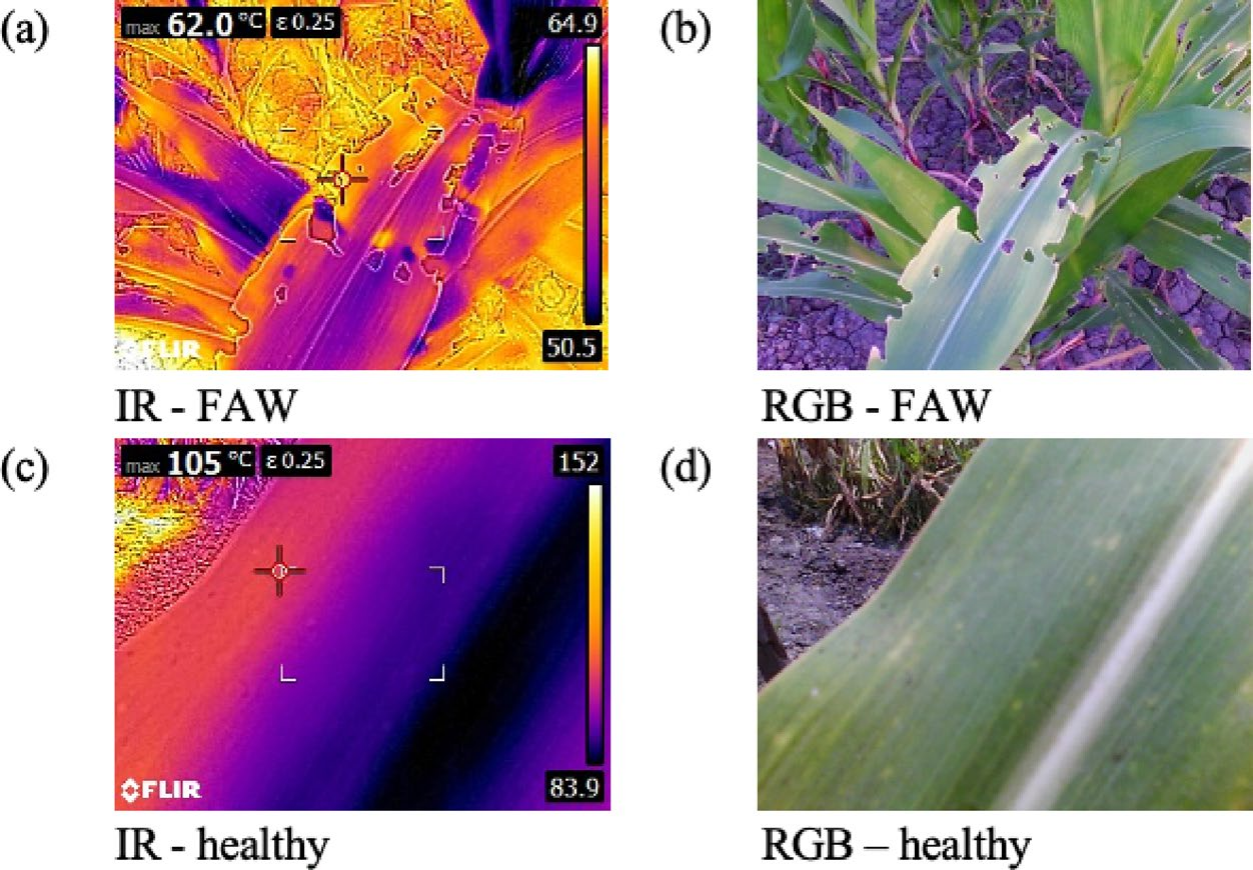
Representative samples from the dataset. (**a**) and (**b**) are the thermal (IR) and RGB images of an FAW infested maize leaf, while (**c**) and (**d**) are the IR and RGB images of a healthy maize leaf.

**Fig. 2:**
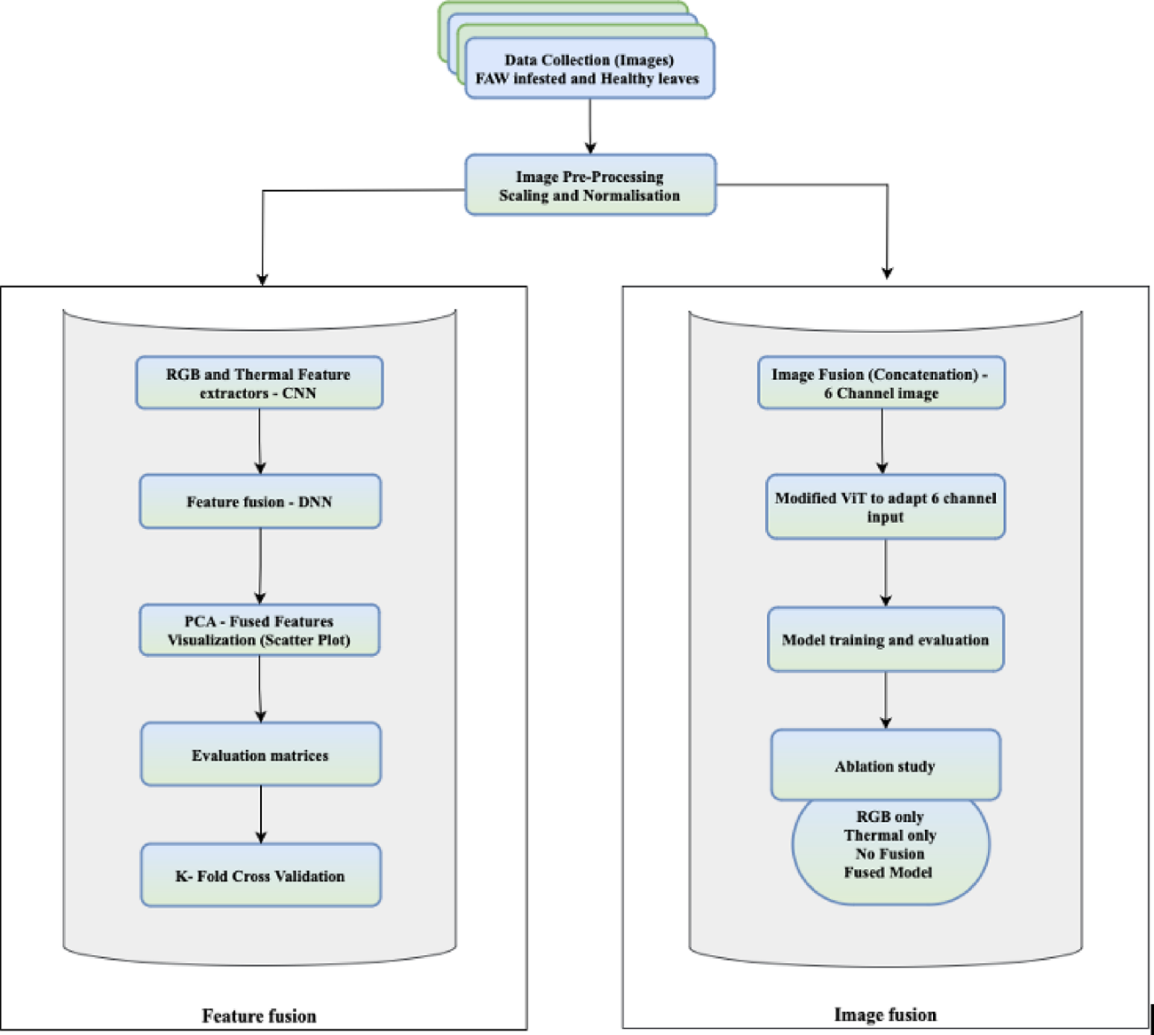
Workflow of the Proposed Network Architecture.

This study utilized paired RGB and thermal images^54^ of Fall Armyworm (FAW)-infested and healthy crops captured using a FLIR-E8 InfraRed Thermometer. Figure 1 represents sample thermal (infrared) and RGB images of dimensions 320 × 240 and 640 × 480, respectively. These images illustrate the visual and thermal differences leveraged in the proposed fusion-based classification model. The images were then resized to 224 × 224 pixels and normalized using pre-trained ViT statistics (mean and standard deviation) without distorting the salient features. The workflow of the proposed architecture is presented in Fig. 2. The dataset comprised 1266 images, with a balanced class distribution of 325 Fall Army Worm (FAW) instances and 308 healthy plant instances, resulting in an approximately 1:1 class ratio. Python version-3.10.3 was used for analysis.

Two complementary fusion strategies were adopted to evaluate the impact of multimodal integration at different stages of the pipeline. Feature level fusion enables interpretability and analysis of learned representations, while image-level fusion through ViT offers an end-to-end learning strategy leveraging attention mechanisms. This dual-path design provides a comprehensive evaluation of fusion strategies.

### Feature extraction and fusion

Separate CNNs were tailored to extract features from each modality. Both networks output a fixed-size feature vector, which is then concatenated to form a fused feature vector. The fused feature vector is passed through a DNN, which learns the relationships between the extracted features. The fusion DNN consists of fully connected layers with ReLU activations and dropout for regularization. This model is capable of learning the combined features from both modalities, improving its ability to distinguish between the two classes. To begin with, the images were resized to fit within a fixed target size of 224 × 224 by a scaling factor:

1 $$scale\_factor = \frac{t\arg et\_size}{{\max \left( {w,h} \right)}}$$

where h and w represents the height and width of the image. Using this factor, the images were resized to new width and height accordingly: $new\_w=int\left(w \times scale\_factor\right), new\_h=int(h \times scale\_factor)$. After resizing, the image was placed on a 224 × 224 canvas using padding or center-cropping to maintain the original aspect ratio. The images were then converted to tensors with shape (C, H, W), where C = 3 for RGB channels, and normalizing the images using a mean μ = [0.485, 0.456, 0.406] and standard deviation σ = [0.229, 0.224, 0.225], which are specific to the ViT model. This normalization follows the formula

2 $$Normalized\_image = \frac{image - \mu }{\sigma }$$

It was verified that each RGB image had a corresponding thermal image. Labels were assigned for each class as 0 for “FAW” or 1 for “Healthy”. For feature extraction, two custom CNN models:RGBFeatureExtractor and ThermalFeatureExtractor and were created for the RGB and thermal images, respectively. The RGB feature extractor used two convolutional layers, followed by max-pooling and a fully connected (FC) layer, mapping the flattened features to a 256-dimensional vector. Similarly, the thermal feature extractor performed operations with two convolutional layers, followed by max-pooling and a final FC layer, producing a 256-dimensional feature vector. The RGB and thermal features were then fused by concatenating them along the feature dimension. 

These fused features were then passed through a DNN consisting of three fully connected layers. The first layer mapped the input size of 512 (since each feature extractor produced 256 features) to a hidden layer of 1024 dimensions. The second layer reduced the dimension to 512, and the third layer outputted two classes for binary classification. During training, the model was optimized using cross-entropy loss for binary classification, with the Adam optimizer and a learning rate of 0.0001. The loss function is defined as

3 $$Loss = - \mathop \sum \limits_{i = 1}^{N} y\_true_{i} \log \left( {y\_pred_{i} } \right)$$

where ${y}_{true}$ represented the true labels and ${y}_{{pred}_{i}}$ represented the predicted probabilities for each class. Accuracy is computed as

4 $$accuracy = \frac{1}{N}\mathop \sum \limits_{i = 1}^{N} \overline{{\underline {\parallel } }} \left( {\widehat{{y_{i} }} = y_{{true_{i} }} } \right)$$

where $\overline{\underline{\parallel }}$ was the indicator function, returning 1 if the prediction was correct and 0 otherwise. This process allowed the model to effectively learn to classify the RGB and thermal image pairs based on their fused features. To visualize the results of a model’s classification, PCA was used to reduce the dimensions of the fused features to 2D for visualization. The pixel values were clipped to the range [0, 1] to ensure that the image data lies within a standard displayable range, for proper visualization. Given a feature matrix F $\epsilon {\mathbb{R}}^{n \times d}$, where n is the number of samples and d is the dimensionality of the features, PCA computes a transformation matrix ${W}_{PCA} \epsilon {\mathbb{R}}^{d \times 2}$ to project the data into 2D:

5 $$F_{reduced} = FW_{PCA} \epsilon {\mathbb{R}}^{n \times 2}$$

This yields the 2D coordinates of the original features, where fused features ${F}_{fused}$ are obtained by concatenating the RGB and thermal features. If ${F}_{rgb} \epsilon {\mathbb{R}}^{n \times {d}_{1}}$ represents the RGB features and ${F}_{thermal} \epsilon {\mathbb{R}}^{n \times {d}_{2}}$ represents the thermal features, the fused feature matrix ${F}_{fused}$ is given by:

6 $$F_{fused} = \left[ {F_{rgb} ,F_{thermal} } \right]\epsilon {\mathbb{R}}^{{n \times \left( {d_{1} + d_{2} } \right)}}$$

**Fig. 3:**
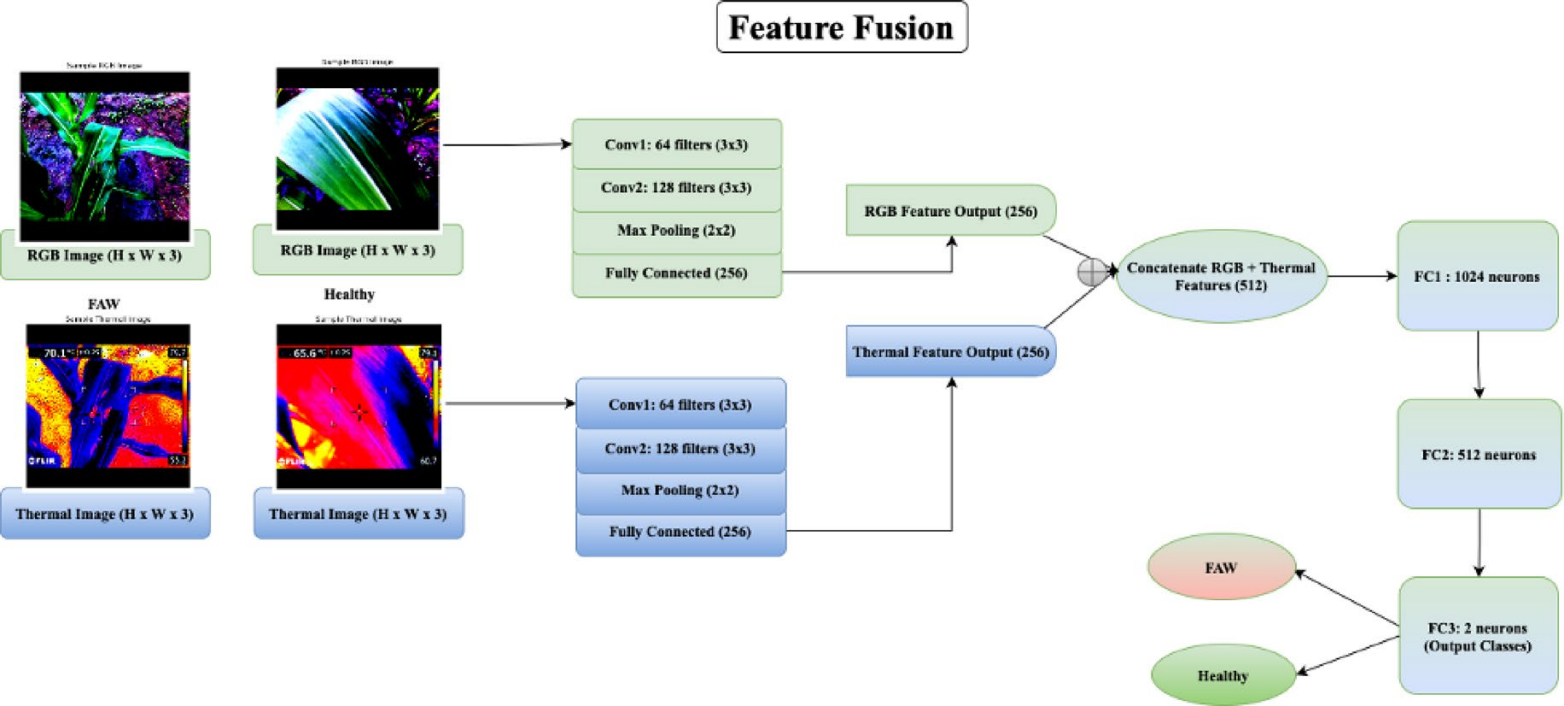
Framework of the Proposed Infrared and Visible Feature Fusion.

The architecture details of the RGB and thermal feature extractors, along with the fusion strategy and training configuration, are summarized in Table 1. This setup was designed to balance representational capacity with computational efficiency, drawing on standard CNN design principles and prior fusion based classification studies. After reducing the features to 2D, a scatter plot is created to visualize the features. For each sample, the 2D reduced feature points ${F}_{reduced} \epsilon {\mathbb{R}}^{n \times 2}$ are plotted, with each point colored according to its class label. Let y ∈ {0,1} represent the class label for each sample. The scatter plot is generated by plotting the i-th sample’s reduced features: Scatter plot of ${F}_{reduced} [\text{i},0]$ vs ${F}_{reduced} [\text{i},1]$ for all samples, colored by ${y}_{i}$. This visualization helps to understand how the model’s fused features are organized in the lower-dimensional space, with distinct classes potentially forming separate clusters. The framework of the proposed feature fusion is presented in Fig. 3. PCA is applied to the feature maps from RGB and thermal images, as well as their fused features, to reduce the dimensionality to 2D for visualization. The key steps includes:

1. *Input Data* The RGB and thermal feature maps, represented as tensors ${X}_{rgb} \epsilon {\mathbb{R}}^{n \times {m}_{rgb}}$ and ${X}_{thermal} \epsilon {\mathbb{R}}^{n \times {m}_{thermal}}$, are extracted from the RGB and thermal images, respectively. The dimensions n, ${m}_{rgb}$ and ${m}_{thermal}$ represent the batch size and the number of features for each modality.
2. *Flattening* The feature maps are then flattened into a 2D matrix:
  7 $$X_{rgb} \epsilon R^{{n \times m_{rgb} }} ,X_{thermal } \epsilon R^{{n \times m_{thermal} }}$$
  where ${m}_{rgb}$ and ${m}_{thermal}$ are the flattened dimensions of the RGB and thermal feature maps.
3. *PCA Application* PCA is applied separately to the flattened RGB and thermal features:
  8 $$X_{rgb,reduced} = PCA\left( {X_{rgb} } \right)$$
  9 $$X_{thermal,reduced} = PCA\left( {X_{thermal} } \right)$$
  This reduces the dimensionality of the feature maps to 2D, making them suitable for visualization.
4. *Fused Feature Maps* A fused feature map was generated by concatenating the RGB and thermal features:
  10 $$X_{fused} = concat\left( {X_{rgb} ,X_{thermal} ,\dim = 1} \right)$$
  The resulting tensor ${X}_{fused}$​ is of the shape (n, ${m}_{rgb}$+ ${m}_{thermal}$).
5. *PCA on Fused Features* PCA is applied to the fused features to reduce them to 2 dimensions:
  11 $$X_{fused,reduced} = PCA\left( {X_{fused} } \right)$$

After PCA, the reduced feature matrix ${X}_{fused, reduced}$, reduced has the shape (n,2), where each row represents a 2D point in the reduced space. After reducing the features to 2D, the visualization is carried out using a scatter plot. Each point in the scatter plot represents a sample (image) in the batch, with the color corresponding to the class label (e.g., “FAW” or “Healthy”).

**Table 1:** CNN architecture and hyperparameter settings for Feature-Level fusion.

Component	Architecture details
Input modality	Paired RGB images and thermal images, resized to 224 × 224 with aspect ratio preserved
CNN for RGB (RGB feature extractor)	2 Conv layers (kernel = 3 × 3, stride = 1, padding-1) Channels: 64 $\to$ 128 MaxPool2d (2 × 2) FC: Output 256-dim
CNN for thermal (Thermal feature extractor)	2 Conv layers (kernel = 3 × 3, stride = 1, padding-1) Channels: 64 $\to$ 128 MaxPool2d (2 × 2) FC: Output 256-dim
Activation function	ReLU after each convolution and fully connected (FC) layer
Fusion method	Concatenation of 256-dim RGB + 256-dim thermal features (fused 512-dim vector)
Fusion DNN	FC1: 512 $\to$ 1024 ReLU + Dropout (p = 0.5) FC2: 1024 $\to$ 512 FC3: 512 $\to$ 2 (Binary Classification)
Optimizer	Adam
Learning rate	0.0001
Loss function	CrossEntropyLoss
Batch size	32
Epochs	10

The trained model was evaluated on a held-out test dataset. Performance was quantified using accuracy, precision, recall, F1-score, and the Area Under the ROC Curve (AUC). The dataset was split into training and test sets, ensuring 80% of the data was used for training and 20% for testing. This split can be represented mathematically as:

$$train\_size = 0.8 \times len(train\_dataset)$$

$$test\_size = len(train\_dataset) - train\_size$$

The model then generated logits (raw scores) for each class, and the predicted class for each sample was obtained by selecting the class with the highest logit score using the operation:

$$preds = argmax(outputs, 1)$$

These predicted labels were compared with the true labels to calculate performance metrics. The accuracy is calculated as:

12 $$Accuracy = \frac{{\mathop \sum \nolimits_{i = 1}^{N} \overline{{\underline {\parallel } }} \left( {\widehat{{y_{i} }} = y_{i} } \right)}}{N}$$

where $\widehat{{y}_{i}}$ is the predicted label for sample i, ${y}_{i}$ is the true label, and $\overline{\underline{\parallel }}(\widehat{{y}_{i}} = {y}_{i})$ is an indicator function that returns 1 if the prediction is correct and 0 otherwise. The precision is calculated as:

13 $$Precision = \frac{True\;Positives}{{True\;Positives + False\;Positives}}$$

where True Positives (TP) are correctly predicted positive samples, and False Positives (FP) are incorrectly predicted positive samples. The recall (or sensitivity) was computed using the formula:

14 $$Recall = \frac{True\;Positives}{{True\;Positives + False\;Negatives}}$$

where False Negatives (FN) are the samples that are actually positive but predicted as negative. The F1-score was computed:

15 $$F1 - Score = 2 \times \frac{Precision \times Recall}{{Precision + Recall}}$$

The AUC (Area Under the ROC Curve) was calculated to evaluate the model’s ability to discriminate between the positive and negative classes across various thresholds. The ROC curve, which displays the true positive rate (TPR) against the false positive rate (FPR), is integrated to determine the AUC. The ROC curve was generated by calculating FPR and TPR for various classification thresholds, and the AUC was computed as the area under the curve:

16 $$AUC = \mathop \smallint \limits_{0}^{1} TPR\left( x \right)dx$$

The final ROC curve, which plots FPR on the x-axis and TPR on the y-axis, was generated, with the diagonal dashed line representing random guessing (AUC = 0.5). The AUC score quantified the overall performance, and the closer the ROC curve was to the top-left corner, the better the model’s ability to distinguish between positive and negative classes at various thresholds. Precision-Recall Curve and Confusion Matrix were then plotted for a more detailed evaluation of the model’s performance. The Precision-Recall Curve visualizes the trade-off between precision and recall for different threshold values of predicted probabilities, which is especially useful for imbalanced datasets. The Confusion Matrix provides a table of correct and incorrect predictions, typically structured for binary classification as:

**Table:** 

	Predicted negative	Predicted positive
Actual negative	True negatives (TN)	False positives (FP)
Actual positive	False negatives (FN)	True positives (TP)

To ensure a robust and reliable evaluation, we employed Stratified K-Fold Cross-Validation. This technique mitigates bias from single train-test splits by partitioning the dataset into K subsets (folds). The model is trained on K-1 folds and tested on the remaining fold, repeating this process K times so each data point is used for testing exactly once. Critically, Stratified K-Fold maintains the original class proportions within each fold, which is especially beneficial for imbalanced datasets. Performance metrics, including Precision, Recall, F1-Score, and ROC-AUC, were calculated for each fold, and the final performance was determined by averaging these metrics across all folds. Following training, the model was evaluated on both the training and testing sets, providing a comprehensive assessment of its performance and generalization capabilities on unseen data.

### Vision transformer (ViT)

**Fig. 4:**
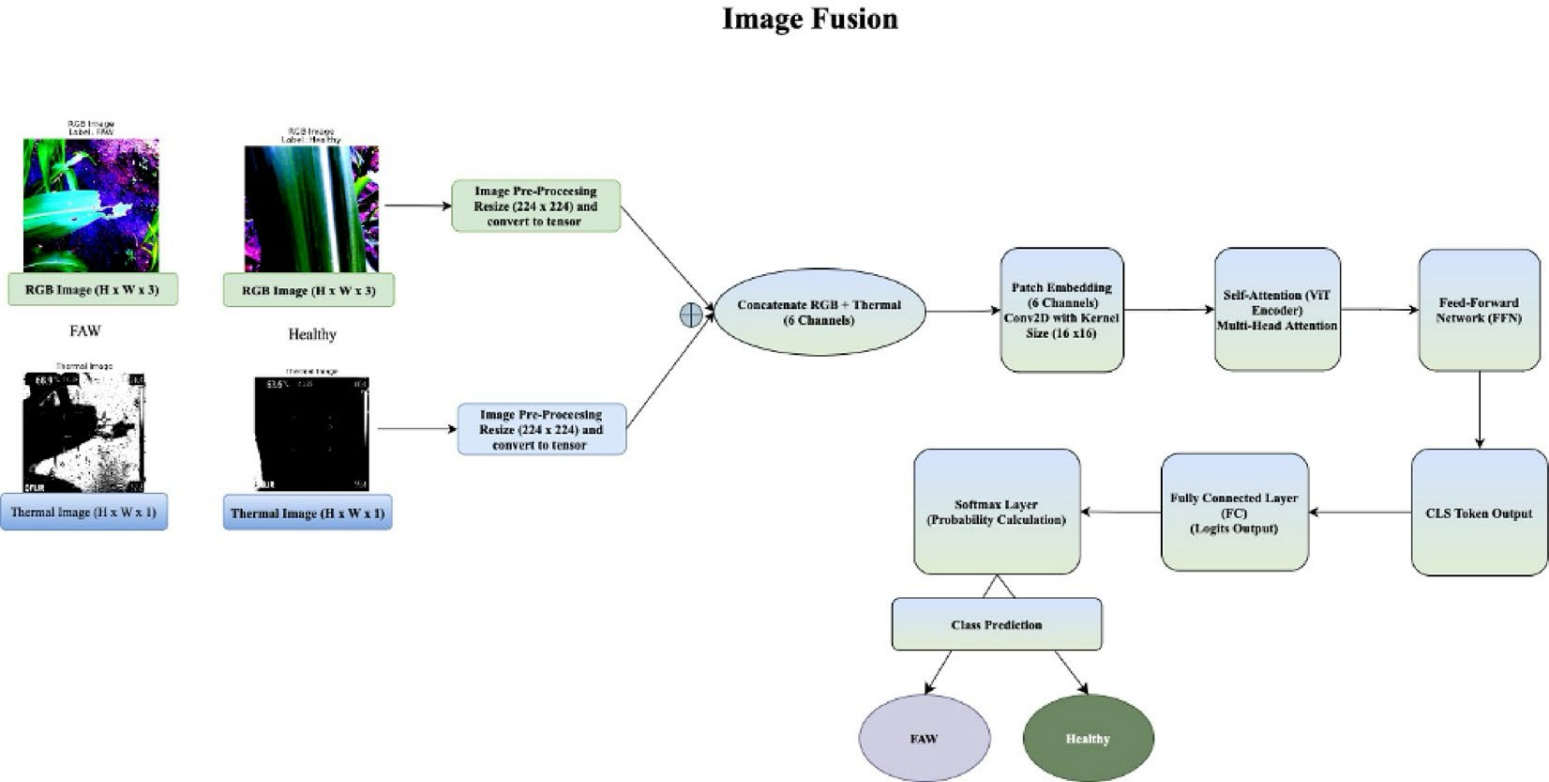
Framework of the Proposed InfraRed and Visible Image Fusion.

A pre-trained ViT model, fine-tuned on the fused features, performed the final classification since the model demonstrates superior performance in image classification tasks and effectively captures long-range dependencies in images. The ViT model was trained using a hybrid image dataset consisting of both RGB and thermal images. This approach utilized a custom dataset that combined these two modalities to create a unified 6-channel input for the modified Vision Transformer model. The framework of the proposed image fusion is presented in Fig. 4. Below is a breakdown of the key methodology and equations involved.

1. *Image Preprocessing* During preprocessing, the pixel values were normalized by subtracting the μ (mean) and dividing by the σ (standard deviation) for each channel c (where c ∈ [1, 6]), ensuring uniform scale across modalities to aid convergence. The thermal channels were normalized similarly to the RGB channels but with their own specific mean and standard deviation values.
2. *Custom Dataset Class* The RGB and thermal images were then concatenated along the channel axis to produce a 6-channel image ${I}_{fused}$. The equation for image fusion is as follows:
  17 $$I_{fused} = \left[ {I_{RGB} ,I_{thermal} } \right]$$
  where ${I}_{RGB}$ is the RGB image with shape (3, H, W) and ${I}_{thermal}$ is the thermal image with shape (3, H, W). The resulting fused image, ${I}_{fused}$, has shape (6, H, W).
3. *Modified Vision Transformer (ViT) Model* The ViT model was adapted to accept 6-channel input images by modifying the input and patch embedding layers. The convolutional embedding operation ($16 \times 16 kernel, stride 16 \times 16)$ generated flattened patches embedded into a high-dimensional space. For an input image I with 6 channels, the patch embedding operation is represented as:
  18 $$I_{patch} = Conv2D\left( {I;6,H,W} \right) \to Flattened\;Patches$$
4. *Training the Model* The model was trained using Cross-Entropy Loss for binary classification (class 0 = “FAW” and class 1 = “Healthy”). The Cross-Entropy Loss is calculated as:
  19 $$L_{CE} = - \left( {y.\log \left( {\hat{y}} \right) + \left( {1 - y} \right).\log \left( {1 - \hat{y}} \right)} \right)$$
  where ${\widehat{m}}_{t}$ and ${\widehat{v}}_{t}$ are biased-corrected first and second moment estimates, respectively, $\eta$ is the learning rate, and $\epsilon$ ensures numerical stability.

where $y$ is the true label (either 0 or 1), and $\widehat{y}$ is the predicted probability of the positive class. The model’s parameters were optimized using the AdamW optimizer, where updates follow:

20 $$\theta_{t} = \theta_{t - 1} - \eta .\frac{{\hat{m}_{t} }}{{\sqrt {\hat{v}_{t} } + \epsilon }}$$

5. *Evaluation* After training, the model is evaluated on the validation dataset. To visualize the results of model’s classification on fused RGB and thermal images, the RGB part of the image is extracted from the first 3 channels (indices 0 - 2), and the thermal part is extracted from the last 3 channels (indices 3 - 5):
  21 $$I_{RGB} = fused\_img\left[ {0:3,:,:} \right]$$
  22 $$I_{thermal} = fused\_img\left[ {3:6,:,:} \right]$$

For visualization, RGB channels and thermal channels were clipped to the range [0,1]. The RGB and thermal images were blended using a weighted sum. The equation for the fused RGB image is:

23 $$fused\_rgb = 0.6 \times I_{RGB} + 0.4 \times I_{thermal}$$

This linear combination gives more weight to the RGB image (0.6) and less to the thermal image (0.4), allowing for the color details from the RGB image to be emphasized while still integrating the thermal information. The model then runs inference on the fused image tensor, outputting a set of logits, which represent the raw, unnormalized scores for each class. These logits are passed through a softmax function to obtain the predicted class label:

24 $$logits = model\left( {I_{fused} } \right)$$

25 $$pred\_label = \arg {\text{max}}\left( {logits} \right)$$

where logits represents the model’s output, and pred_label is the index of the class with the highest score (either 0 for “FAW” or 1 for “Healthy”). The visualization then displays three panels: the raw RGB image, the raw thermal image (shown with a “hot” colormap), and the fused image with the predicted label superimposed on it. The predicted label is specifically displayed in the fused image panel. This visualization process aids in understanding how the model classifies fused images and provides insight into the combination of RGB and thermal modalities in the image processing pipeline.

### Ablation study

An ablation study was conducted to evaluate image fusion performance using metrics such as accuracy, precision, recall, F1-score, confusion matrix, ROC-AUC, and precision-recall curves. For the model configurations in the ablation study, the experiment types include: (1) RGB only, using RGB features extracted by the RGBFeatureExtractor; (2) Thermal only, using thermal features extracted by the ThermalFeatureExtractor; (3) No Fusion, using both RGB and thermal features without fusion; and (4) ViT with Fusion, using the Vision Transformer (ViT) for fusion of RGB and thermal images.

The proposed architecture integrates two complementary fusion strategies: *feature-level fusion* and *image-level fusion*, both applied to the same paired RGB and IR dataset for classifying FAW-infested and healthy maize leaves. In the feature level approach RGB and thermal features were processed through separate CNN-based feature extractors that produced 256-dimensional feature vectors each. These were concatenated into a fused vector passed through a DNN comprising fully connected layers. This allowed discriminating between classes using combined spatial-spectral patterns. PCA based visualization provided interpretability by showing class separation in the latent space.

In parallel, the image-level fusion strategy concatenated RGB and thermal images into a 6-channel input fed to a modified ViT, capable of capturing local and global relationships via self-attention. The fused model was trained to minimize classification loss and evaluated using accuracy, precision, recall, F1-score and AUC. An ablation study is also conducted to compare the performance of the fused model with RGB-only, thermal-only and unfused inputs. While both pipelines differ in architecture, they share a common goal: learning the distinguishing characteristics of healthy and FAW infested leaves by leveraging complementary spectral information. The dual path strategy provided complementary advantages: feature-level interpretability and image-level robustness, forming an integrated system optimized for practical pest detection in agricultural environments.

## Results

**Fig. 5:**
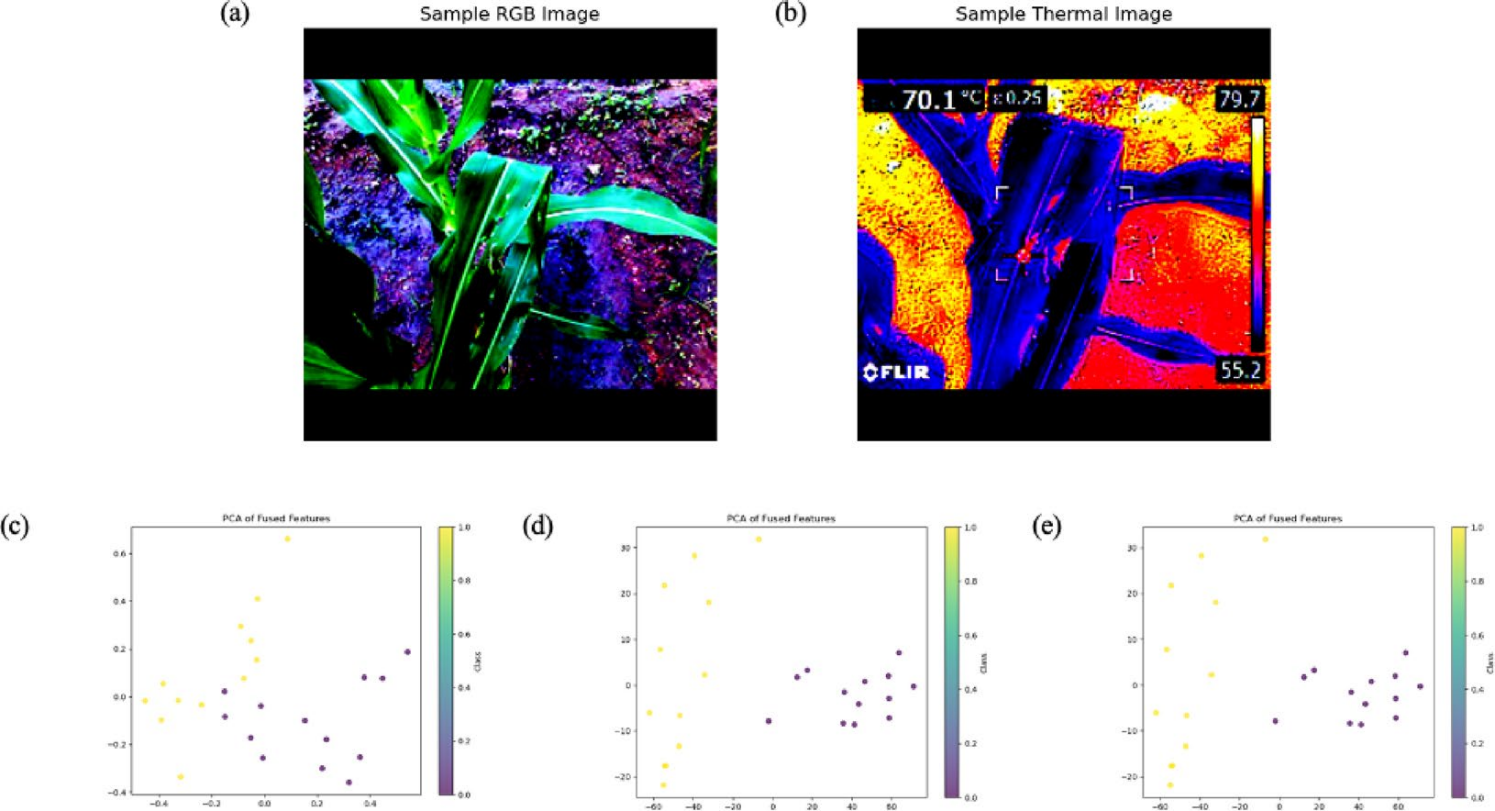
(**a**) and (**b**) represent the Sample RGB and Thermal Image of FAW class; while (**c**), (**d**) and (**e**) are the visualization of fused features by PCA representing the first, second and third principal component respectively.

**Fig. 6:**
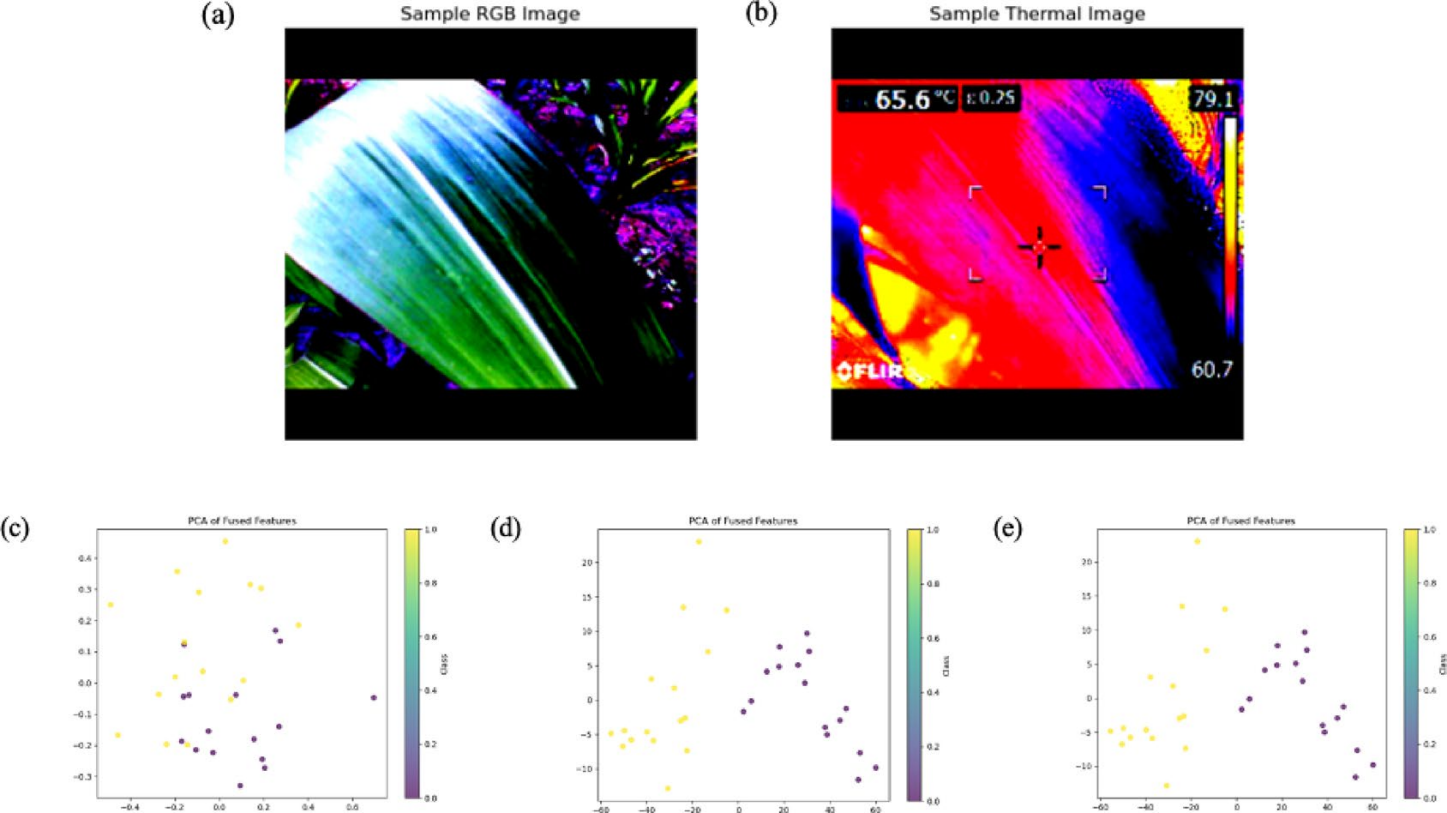
(**a**) and (**b**) represent the Sample RGB and Thermal Image of healthy class; while (**c**), (**d**) and (**e**) are the visualization of fused features by PCA representing the first, second and third principal component respectively.

The results of the model’s feature extraction and fusion process demonstrate how the shape of feature maps evolves through different stages of the network. For the RGB and thermal images, the feature maps are extracted through separate model pathways. After passing through a convolutional network (CNN), an RGB image with an input size of 3 × 224 × 224 produces a feature map with the shape (batch_size, 256, 7, 7), where 256 represents the number of channels, and 7 × 7 is the reduced spatial resolution. Similarly, the thermal image yields a feature map with fewer channels or a different resolution, such as (batch_size, 128, 7, 7). Once the features from both the RGB and thermal images are extracted, they are fused by concatenating them along the channel dimension, resulting in a combined feature map of shape (batch_size, 384, 7, 7). This fusion process integrates the information from both modalities, significantly enhancing the model’s ability to make predictions based on combined RGB and thermal inputs. These fused features are then further processed by dimensionality reduction for visualization and classification. The fusion of these features and their subsequent transformation through the model highlights the importance of multi-modal learning, where the combination of RGB and thermal features leads to a richer and more comprehensive representation of the input data. Principal Component Analysis (PCA) of the fused features reveals a nuanced pattern of class separability as represented in Figs. 5 and 6.

The first principal component, which accounts for a substantial portion of the data variance, demonstrates some overlap between classes, suggesting that the original features may not be sufficient to clearly distinguish between them. However, the second and third principal components show strong class separation, indicating that the fused features, when projected onto these dimensions, are highly discriminative. Interestingly, the near-identical plots for the second and third components imply that most of the class-separating information is captured within the first two principal components. This suggests that the fused feature representation, after dimensionality reduction through PCA, provides a more concise and informative view of the data, effectively highlighting key differences between the classes while mitigating noise or redundancy in the original features.

The wide range observed in the first principal component plot likely reflects the inherent variability within the raw, untransformed fused features, as PCA prioritizes maximum-variance capture. In contrast, the narrower range and high similarity between the second and third principal components suggest that after the initial variance captured by the first component, the remaining variance is minimal. This could be attributed prior data normalization or limited inherent separability between the classes within the fused feature space.

**Fig. 7:**
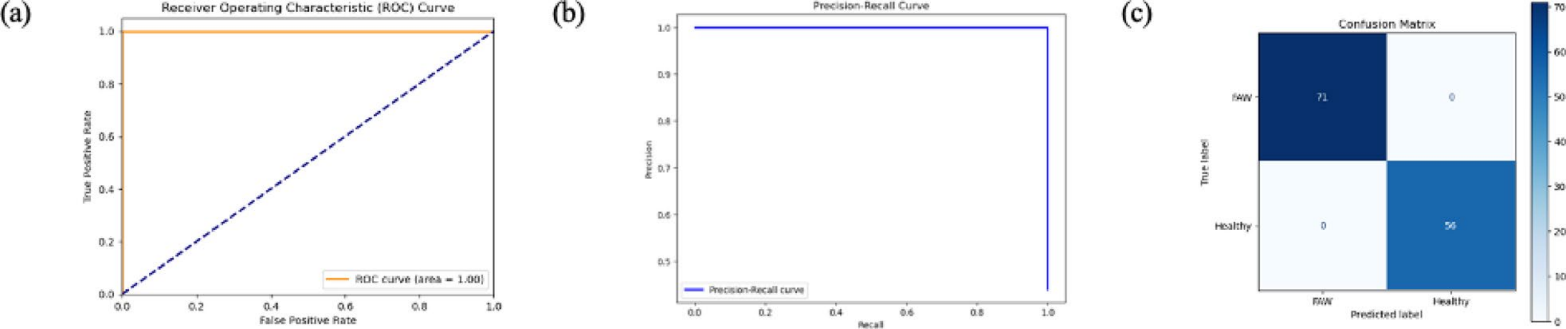
ROC Curve, Precision-Recall Curve and Confusion Matrix evaluated for the Feature Fusion Methodology.

The feature-level fusion of RGB and thermal images for Fall Army Worm (FAW) detection demonstrated exceptional performance, achieving near-perfect accuracy, precision, recall, F1-score, and AUC scores across training, testing, and K-Fold Cross-Validation sets, as given in Table 2. PCA visualization revealed clear separation between FAW-infested and healthy leaves in the feature space. Figure 7 shows ROC and Precision-Recall (PR) curves along with the confusion matrix for the fused model. The ROC and PR curves approach the ideal top-left corners, suggesting near-perfect classification. The confusion matrix confirms this, with no misclassifications recorded in the test set, validating the model’s precision and reliability. These results strongly demonstrate that combining RGB and thermal data provides a robust and effective approach for accurate FAW detection.

**Table 2:** Performance Metrics evaluated for the Feature-Fusion Methodology.

	Accuracy	Precision	Recall	F1–Score	AUC Score
Train	1.00	1.00	1.00	1.00	1.00
Test	1.00	1.00	1.00	1.00	1.00
K–Fold Cross Validation					
Train	0.99	0.98	0.99	0.99	0.99
Test	0.99	0.98	1.00	0.99	0.99

**Fig. 8:**
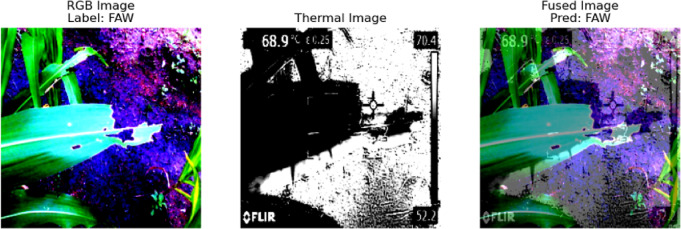
Visual representation of Image Fusion for FAW class.

**Fig. 9:**
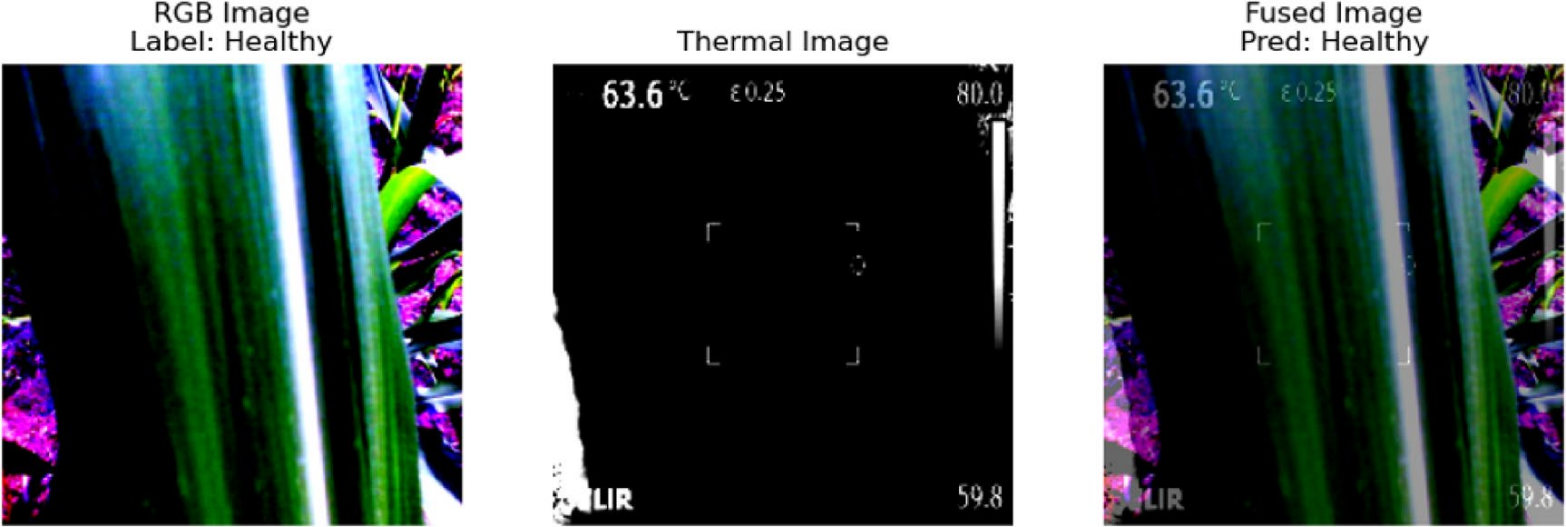
Visual representation of Image Fusion for Healthy class.

The Image-fusion model significantly outperformed the RGB-only and thermal-only models across all metrics. It achieved higher accuracy, precision, recall, and F1-score, with a superior ROC-AUC, demonstrating improved discrimination between FAW and healthy classes. Figures 8 and 9 depict the visual outputs of the image-fusion process for FAW and healthy samples, respectively. These examples highlight how image fusion enriches detail from both modalities, improving visual interpretability and decision-making for both automated and manual analysis.

**Fig. 10:**
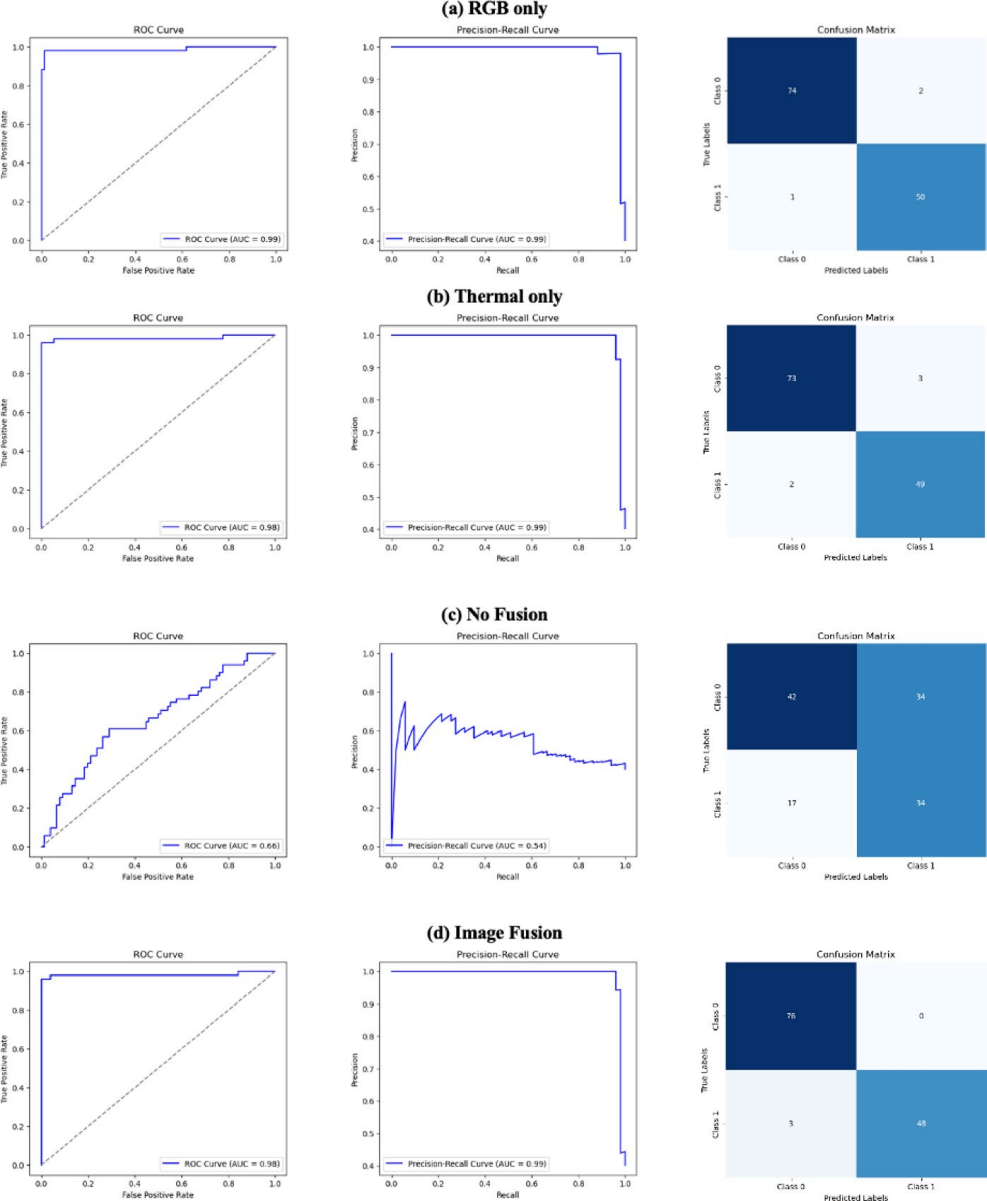
Ablation Study—ROC Curve, Precision-Recall Curve and Confusion Matrix evaluated for the Image Fusion Methodology ((**a**) RGB only, (**b**) Thermal only, (**c**) No Fusion, (**d**) Image Fusion).

The RGB-only model performed reasonably well, but not as effectively as the fused model. The thermal-only model, while useful for detecting heat signatures, was less accurate than the fused approach, indicating that thermal images alone are less informative. The no-fusion scenario performed worse than both the individual modalities and fused models. The fused model clearly outperformed all others, indicating the benefit of interactive feature learning. Table 3 presents a comparison of results from all configurations. The fused model’s confusion matrix showed fewer false positives and negatives, confirming more accurate predictions. The classification report further validated higher precision, recall, and F1-score, particularly for the FAW class. Figure 10 presents ROC, PR curves and confusion matrices for the RGB-only, thermal-only, no-fusion and fused models. The fused model consistently outperforms all others in both curve profiles and quantitative metrics.

**Table 3:** Performance Metrics evaluated for the Image-Fusion Methodology using Ablation study.

		RGB only	Thermal only	No fusion	Fused model
Accuracy	Train	0.98	0.99	0.67	0.99
	Test	0.98	0.96	0.60	0.98
Precision		0.98	0.96	0.63	0.98
Recall		0.98	0.96	0.60	0.98
F1–Score		0.98	0.96	0.60	0.98
AUC–ROC		0.99	0.98	0.67	0.98

## Discussion

The results of this study demonstrate the effectiveness of hybrid DNN-ViT architecture for accurately detecting FAW infestations in maize leaves using fused RGB and IR images. The model integrates two complementary fusion strategies: feature-level fusion using CNNs and a DNN, and image-level fusion using a modified ViT. The combined framework achieved near-perfect performance across multiple evaluation metrics, indicating that complementary information from RGB and thermal modalities provide a robust and informative representation of leaf health. The multimodal input enabled the model to distinguish between healthy and infested leaves, even under varied imaging conditions. The ViT component played a critical role in end-to-end learning, with its attention mechanism effectively capturing both local and global features from the fused input, outperforming conventional CNN-based architectures in accuracy, recall, and AUC. In Parallel, the feature-level fusion branch enabled interpretability through PCA-based visualization of the fused feature space, which revealed well-separated clusters for individual classes, confirming strong intra-class compactness and inter-class separability. While the proposed model demonstrated strong performance, certain limitations remain. The dataset size and environmental diversity were limited, which may affect generalization. Moreover, the registration-free fusion strategy performs optimal only when images are captured using sensors that inherently provide aligned RGB-thermal outputs, such as the FLIR infrared thermometer used in this study. Future work will focus on expanding the dataset across varied crop conditions, exploring adaptive fusion strategies for unaligned inputs and validating model generalizability.

## Conclusion

This study presented a novel deep learning framework integrating feature-level fusion of RGB and thermal images using a hybrid DNN-ViT architecture for the classification of FAW-infested and healthy maize crops. The proposed approach effectively combines the complementary strengths of visible and thermal modalities, yielding significantly enhanced classification performance. Experimental results showed that the fused model outperformed individual RGB-only and thermal-only models, achieving a test accuracy of 0.98 along with high precision, recall, F1-score, and AUC-ROC values. The ablation study further confirmed the benefit of multimodal image fusion in capturing discriminative features necessary for accurate FAW detection. The key contribution of this work lies in developing a registration-free feature-level fusion strategy using a hybrid DNN-ViT architecture designed for effective operation under real-world agricultural conditions. This advancement offers a scalable and robust solution for automated pest monitoring to support precision agriculture. Future research will explore advanced fusion techniques, evaluating model robustness under varying environmental conditions, and deploy the system in field settings to assess its practical impact and usability.

## Supplementary Information

Below is the link to the electronic supplementary material.

Supplementary Material 1

## Data Availability

The dataset has been made publicly available in the Figshare Data repository as a part of a peer reviewed data publication^54^. Detailed information on data acquisition, sensor specifications, environmental conditions and annotation protocols is provided in the associated data article. The dataset can be accessed at: https://figshare.com/s/677d2384ba6e02db9230 (10.6084/m9.figshare.28388018).

## Code availability

The custom python code developed for this study is available as supplementary file (“Supplementary Code.zip”) and includes all scripts necessary to reproduce the multimodal feature fusion, image-level fusion and ablation experiments described in the manuscript. The dataset used is publicly available on Figshare. All dependencies are listed within the code file. Readers can execute the python script to reproduce the reported results.

## References

[1] Wang, C. et al. A two-stream network with complementary feature fusion for pest image classification. *Eng. Appl. Artif. Intell.* **124**, 106563. 10.1016/j.engappai.2023.106563 (2023).

[2] Tang, Z., Chen, Z., Qi, F., Zhang, L. and Chen, S. Pest-YOLO: Deep Image Mining and Multi-Feature Fusion for Real-Time Agriculture Pest Detection. In *2021 IEEE International Conference on Data Mining (ICDM)*. 1348–1353. 10.1109/ICDM51629.2021.00169. (IEEE, 2021)

[3] Liu, S., Lan, X., Chen, W., Zhang, Z. & Qiu, C. AGMFusion: A Real-time end-to-end infrared and visible image fusion network based on adaptive guidance module. *IEEE Sens. J.* **24**(17), 28338–28350. 10.1109/JSEN.2024.3426274 (2024).

[4] Xu, M., Chen, H. and Varshney, P.K. A novel approach for image fusion based on Markov Random Fields. In *2008 42nd Annual Conference on Information Sciences and Systems*. 344–349. 10.1109/CISS.2008.4558549. (IEEE, 2008)

[5] Aishwarya, N. & Bennila Thangammal, C. An image fusion framework using morphology and sparse representation. *Multimed. Tools Appl.* **77**(8), 9719–9736. 10.1007/s11042-017-5562-4 (2018).

[6] Chen, Y., Cheng, L., Wu, H., Mo, F. & Chen, Z. Infrared and visible image fusion based on iterative differential thermal information filter. *Opt. Lasers Eng.* **148**, 106776. 10.1016/j.optlaseng.2021.106776 (2022).

[7] Li, H., Qiu, H., Yu, Z. & Zhang, Y. Infrared and visible image fusion scheme based on NSCT and low-level visual features. *Infrared Phys. Technol.* **76**, 174–184. 10.1016/j.infrared.2016.02.005 (2016).

[8] Wang, L.-F., Xin, L.-P., Yu, B., Ju, L. & Wei, L. A novel method for determination of the oil slick area based on visible and thermal infrared image fusion. *Infrared Phys. Technol.* **119**, 103915. 10.1016/j.infrared.2021.103915 (2021).

[9] Yang, G., Li, J., Lei, H. and Gao, X. A Multi-scale Information Integration Framework for Infrared and Visible Image Fusion. Preprint at https://arxiv.org/abs/2312.04328. (2024)

[10] M. Chen, Y. Cheng, X. He, X. Wang, Y. Aze, and J. Xiang, “SimpleFusion: A Simple Fusion Framework for Infrared and Visible Images. Preprint at https://arxiv.org/abs/2406.19055. (2024)

[11] Cheng, C., Sun, C., Sun, Y. & Zhu, J. StyleFuse: An unsupervised network based on style loss function for infrared and visible image fusion. *Signal Process. Image Commun.* **106**, 116722. 10.1016/j.image.2022.116722 (2022).

[12] Civardi, G. L. et al. Generation of fused visible and thermal-infrared images for uncooperative spacecraft proximity navigation. *Adv. Space Res.* **73**(11), 5501–5520. 10.1016/j.asr.2023.03.022 (2024).

[13] Sun, J., Zhu, H., Xu, Z. & Han, C. Poisson image fusion based on Markov random field fusion model. *Inf. Fusion* **14**(3), 241–254. 10.1016/j.inffus.2012.07.003 (2013).

[14] Sharma, A. & Saroliya, A. A brief review of different image fusion algorithm. *Int. J. Sci. Res.* **4**(6), 2650–2652 (2013).

[15] Chen, J., Li, X., Luo, L., Mei, X. & Ma, J. Infrared and visible image fusion based on target-enhanced multiscale transform decomposition. *Inf. Sci.* **508**, 64–78. 10.1016/j.ins.2019.08.066 (2020).

[16] Xu, H., Gong, M., Tian, X., Huang, J. & Ma, J. CUFD: An encoder–decoder network for visible and infrared image fusion based on common and unique feature decomposition. *Comput. Vis. Image Underst.* **218**, 103407. 10.1016/j.cviu.2022.103407 (2022).

[17] G. Prema and S. Arivazhagan. NSCT decomposition and VGG-Net based multimodal Infrared and Visible Image Fusion. In *2024 International Conference on Integrated Circuits and Communication Systems (ICICACS)*. 1–6. 10.1109/ICICACS60521.2024.10498702. (IEEE, 2024)

[18] Wu, P., Jiang, L., Li, Y., Fan, H. & Li, J. SFPN: Segmentation-based feature pyramid network for multi-focus image fusion. *Multimed. Tools Appl.* **83**(7), 20055–20082. 10.1007/s11042-023-15342-9 (2023).

[19] Asefpour Vakilian, A. & Saradjian, M. R. An object-based sparse representation model for spatiotemporal image fusion. *Sci. Rep.* **12**(1), 5021. 10.1038/s41598-022-08728-6 (2022). 35322054 10.1038/s41598-022-08728-6 PMC8943014

[20] Hu, H., Wang, X. & Li, T. RETRACTED: Infrared and visible image fusion method based on full convolutional network (FCN). *IFS* **46**(1), 2825–2834. 10.3233/JIFS-236094 (2024).

[21] Liu, Y., Li, X., Liu, Y. & Zhong, W. SimpliFusion: A simplified infrared and visible image fusion network. *Vis. Comput.*10.1007/s00371-024-03423-1 (2024).

[22] Alexander, Q. G., Hoskere, V., Narazaki, Y., Maxwell, A. & Spencer, B. F. Fusion of thermal and RGB images for automated deep learning based crack detection in civil infrastructure. *AI Civ. Eng.* **1**(1), 3. 10.1007/s43503-022-00002-y (2022).

[23] Cai, Z. et al. CMFuse: Cross-modal features mixing via convolution and MLP for Infrared and visible image fusion. *IEEE Sens. J.* **24**(15), 24152–24167. 10.1109/JSEN.2024.3410387 (2024).

[24] C. G, B. J, V. K. B, and S. K. P, “Infrared and Visible Image Fusion using Enhanced Thermal Image. In *2023 International Conference on Intelligent Systems for Communication, IoT and Security (ICISCoIS)*. 392–397. 10.1109/ICISCoIS56541.2023.10100444. (2023)

[25] Ma, J., Yu, W., Liang, P., Li, C. & Jiang, J. FusionGAN: A generative adversarial network for infrared and visible image fusion. *Inf. Fusion* **48**, 11–26. 10.1016/j.inffus.2018.09.004 (2019).

[26] Y. Zhang, Y. Fang, and Q. Zhang, “Focus Fusion Network for Visible and Infrared Image Fusion. In *ICASSP 2024—2024 IEEE International Conference on Acoustics, Speech and Signal Processing (ICASSP)*. 3850–3854. 10.1109/ICASSP48485.2024.10445881. (IEEE, 2024)

[27] Zhang, Z., Wu, X. & Xu, T. FPNFuse: A lightweight feature pyramid network for infrared and visible image fusion. *IET Image Process.* **16**(9), 2308–2320. 10.1049/ipr2.12473 (2022).

[28] Mogan, J. N., Lee, C. P. & Lim, K. M. Ensemble CNN-ViT using feature-level fusion for gait recognition. *IEEE Access* **12**, 108573–108583. 10.1109/ACCESS.2024.3439602 (2024).

[29] Lu, X., Xu, Y. & Yuan, W. PDRF-Net: A progressive dense residual fusion network for COVID-19 lung CT image segmentation. *Evol. Syst.* **15**(2), 267–283. 10.1007/s12530-023-09489-x (2024). 10.1007/s12530-023-09489-x PMC9936947 38625320

[30] Long, X. & Sun, J. Image segmentation based on the minimum spanning tree with a novel weight. *Optik* **221**, 165308. 10.1016/j.ijleo.2020.165308 (2020).

[31] Li, J., Sun, S., Li, S. & Xia, R. CascadeMedSeg: Integrating pyramid vision transformer with multi-scale fusion for precise medical image segmentation. *SIViP* **18**(12), 9067–9079. 10.1007/s11760-024-03530-5 (2024).

[32] Si, H. et al. A registration algorithm for the infrared and visible images of apple based on active contour model. *Vis. Comput.* **40**(4), 2833–2855. 10.1007/s00371-023-02989-6 (2024).

[33] Wang J. & Jiang M. TEPFusion: An End-to-End Infrared and Visible Image Fusion Network Based on Transformer and Progressive Channel Fusion. In *2024 5th International Conference on Electronic Communication and Artificial Intelligence (ICECAI)*. 60–65. 10.1109/ICECAI62591.2024.10674894. (IEEE, 2024)

[34] Kalamkar, S. & Amalanathan, G. M. MDA-ViT: Multimodal image fusion using dual attention vision transformer. *Multimed. Tools Appl.*10.1007/s11042-024-19968-1 (2024).

[35] Li, H., Liu, J., Zhang, Y. & Liu, Y. A Deep learning framework for infrared and visible image fusion without strict registration. *Int. J. Comput. Vis.* **132**(5), 1625–1644. 10.1007/s11263-023-01948-x (2024).

[36] Sun, C., Zhang, C. & Xiong, N. Infrared and visible image fusion techniques based on deep learning: A review. *Electronics* **9**(12), 2162. 10.3390/electronics9122162 (2020).

[37] Medalla, J. V. T. Application of Wavelet Technique in Image Fusion and its Introduction as an Early Detection Tool for Spreading of Plant Pests in Philippines’ Agricultural Sector: Initial Stage. In *2018 IEEE 10th International Conference on Humanoid, Nanotechnology, Information Technology,Communication and Control, Environment and Management (HNICEM).* 1–8. 10.1109/HNICEM.2018.8666424. (IEEE 2018)

[38] Upadhyay, N. & Gupta, N. Detecting fungi-affected multi-crop disease on heterogeneous region dataset using modified ResNeXt approach. *Environ. Monit. Assess.* **196**(7), 610. 10.1007/s10661-024-12790-0 (2024). 38862723 10.1007/s10661-024-12790-0

[39] Upadhyay, N. & Gupta, N. Diagnosis of fungi affected apple crop disease using improved ResNeXt deep learning model. *Multimed. Tools Appl.* **83**(24), 64879–64898. 10.1007/s11042-023-18094-8 (2024).

[40] Upadhyay, N. & Gupta, N. SegLearner: A segmentation based approach for predicting disease severity in infected leaves. *Multimed. Tools Appl.*10.1007/s11042-025-20838-7 (2025).

[41] S. Solanki, S. Singh Chouhan, A. Dwivedi, U. P. Singh, and R. K. Patel. Leveraging Deep Learning for the Identification and Categorization of Fruit Diseases. In *2024 IEEE International Conference on Intelligent Signal Processing and Effective Communication Technologies (INSPECT)*. 1–6. 10.1109/INSPECT63485.2024.10896118. (2024)

[42] Chen, Y. et al. Image fusion with sparse representation: A novel local contrast-based preprocessing strategy. *IEEE Sens. Lett.* **6**(5), 1–4. 10.1109/LSENS.2022.3170744 (2022).

[43] Hayes, A.E., Montagna, R. and Finlayson, G.D. New applications of Spectral Edge image fusion. In SPIE Defense + Security, M. Velez-Reyes and D. W. Messinger, Eds., Baltimore, Maryland, United States, May 2016, p. 984009. 10.1117/12.2223703.

[44] Tang, H., Qian, Y., Xing, M., Cao, Y. & Liu, G. MPCFusion: Multi-scale parallel cross fusion for infrared and visible images via convolution and vision transformer. *Opt. Lasers Eng.* **176**, 108094. 10.1016/j.optlaseng.2024.108094 (2024).

[45] Ma, J., Zhang, H., Shao, Z., Liang, P. & Xu, H. GANMcC: A generative adversarial network with multiclassification constraints for infrared and visible image fusion. *IEEE Trans. Instrum. Meas.* **70**, 1–14. 10.1109/TIM.2020.3038013 (2021).33776080

[46] C. Arnold, P. Jouvet, and L. Seoud. SwinFuSR: An image fusion-inspired model for RGB-guided thermal image super-resolution. In *2024 IEEE/CVF Conference on Computer Vision and Pattern Recognition Workshops (CVPRW)*. 3027–3036. 10.1109/CVPRW63382.2024.00308. (IEEE, 2024)

[47] Gallagher, J. E. & Oughton, E. J. Assessing thermal imagery integration into object detection methods on air-based collection platforms. *Sci. Rep.* **13**(1), 8491. 10.1038/s41598-023-34791-8 (2023). 37231037 10.1038/s41598-023-34791-8 PMC10212993

[48] Li, X. et al. RITFusion: Reinforced interactive transformer network for infrared and visible image fusion. *IEEE Trans. Instrum. Meas.* **73**, 1–16. 10.1109/TIM.2023.3342223 (2024).

[49] S. Marappan, P. Kuppusamy, R. John, and S. V. Natesan. A Novel Approach to Preserve Small Scale Details in Fused Image using Guide Filter with NSCT for Visual and Infrared Images. In *2020 2nd International Conference on Computer and Information Sciences (ICCIS)*. 1–5. 10.1109/ICCIS49240.2020.9257704. (IEEE, 2020).

[50] Zhao, F., Zhao, W. & Lu, H. Interactive feature embedding for infrared and visible image fusion. *IEEE Trans. Neural Netw. Learn. Syst.* **35**(9), 12810–12822. 10.1109/TNNLS.2023.3264911 (2024). 37040245 10.1109/TNNLS.2023.3264911

[51] Chouhan, S. S., Kaul, A. & Singh, U. P. Image segmentation using fuzzy competitive learning based counter propagation network. *Multimed. Tools Appl.* **78**(24), 35263–35287. 10.1007/s11042-019-08094-y (2019).

[52] Chouhan, S. S., Patel, R. K., Singh, U. P. & Tejani, G. G. Integrating drone in agriculture: Addressing technology, challenges, solutions, and applications to drive economic growth. *Remote Sens. Appl. Soc. Environ.* **38**, 101576. 10.1016/j.rsase.2025.101576 (2025).

[53] Chouhan, S. S., Singh, U. P. & Jain, S. Performance evaluation of different deep learning models used for the purpose of healthy and diseased leaves classification of Cherimoya (*Annona cherimola*) plant. *Neural Comput. Applic.* **37**(6), 4531–4544. 10.1007/s00521-024-10830-x (2025).

[54] Sandhya, P., Venkataramana, B., Kumar, T. P. & Sujatha, R. Thermal and RGB image dataset for detection and management of Fall Army Worm (FAW) infestation in maize. *Front. Agron. Aug.*10.3389/fagro.2025.1629681 (2025).

